# Supplementary material for: SLAMF7 and IL-6R define distinct cytotoxic versus helper memory CD8+ T cells
Source: Nat Commun. 2020 Dec 11;11:6357. doi: 10.1038/s41467-020-19002-6 (PMC7733515; doi:10.1038/s41467-020-19002-6)
Supplement: Supplementary file 1 — Supplementary Information [file 41467_2020_19002_MOESM1_ESM.pdf]

Supplementary Figures Loyal et al.

SLAMF7 and IL-6R define distinct cytotoxic versus helper memory CD8^{+} T cells

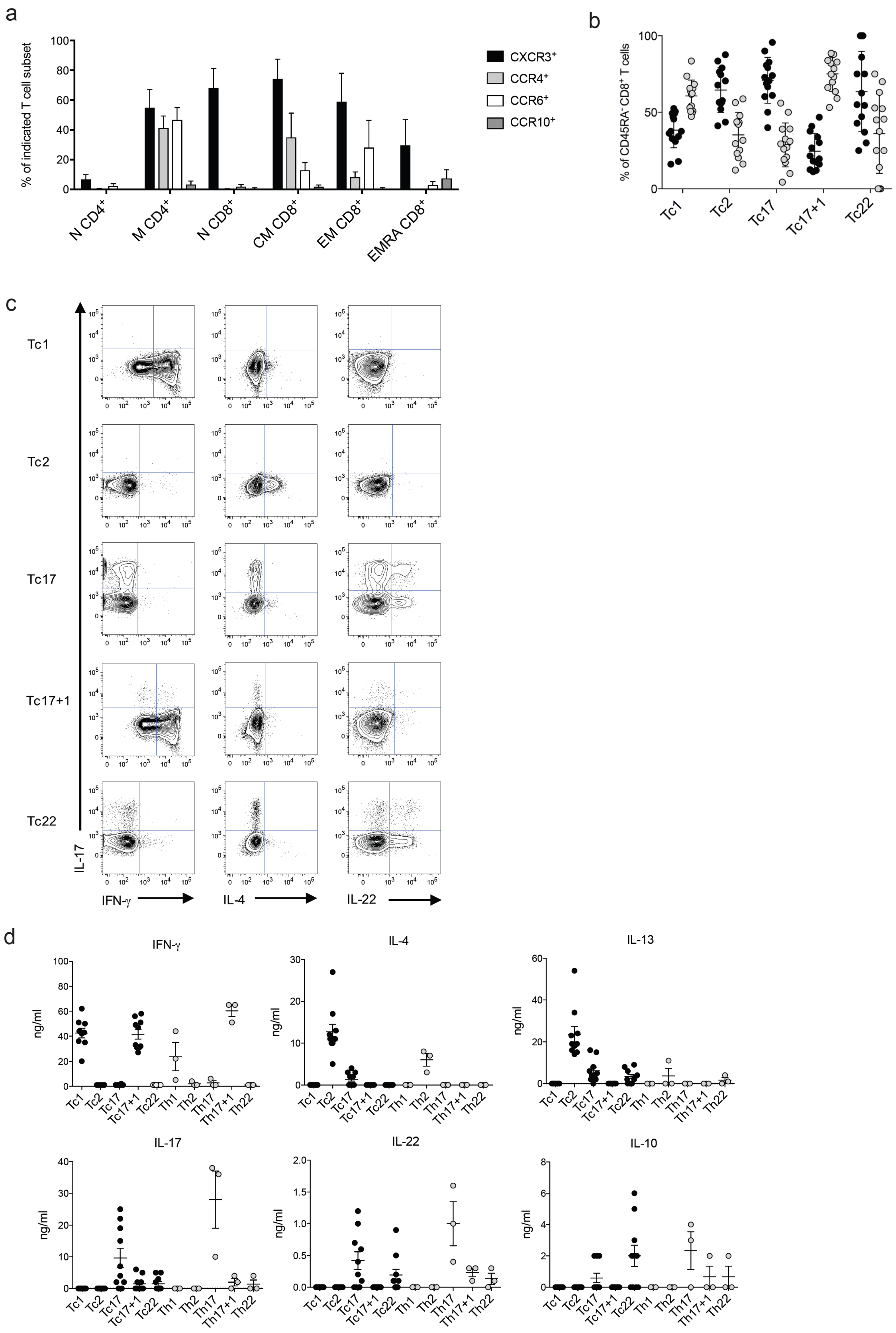
a
% of indicated T cell subset
CXCR3+
CCR4+
CCR6+
CCR10+
N CD4+
M CD4+
N CD8+
CM CD8+
EM CD8+
EMRA CD8+
b
% of CD45RA- CD8+ T cells
CM
EM
Tc1
Tc2
Tc17
Tc17+1
Tc22
c
Tc1
Tc2
Tc17
Tc17+1
Tc22
IL-17
IFN-γ
IL-4
IL-22
d
IFN-γ
IL-4
IL-13
IL-17
IL-22
IL-10
ng/ml
Tc1
Tc2
Tc17
Tc17+1
Tc22
Th1
Th2
Th17
Th17+1
Th22

Supplementary Figure 1: Chemokine receptor distribution and T-cell subset characterizations

a, Distribution of chemokine receptors among naïve and memory (M) $CD4^+$ T cells and naïve (N), effector memory (EM), central memory (CM) and effector-memory with reacquired CD45RA expression (EMRA) $CD8^+$ T cells (*n* = 28), mean ± SEM. **b,** Frequencies of $CCR7^+$ T_{CM} (black circles) and $CCR7^-$ T_{EM} (grey circles) $CD45RA^-$ memory cells among gated Tc-cell subsets (*n* = 14). Mean ± SEM. **c,** Representative FACS plots of sorted and polyclonal activated Tc-cell subsets. **d,** Sorted Tc- (black circles) and Th- (grey circles)cell subsets were stimulated for 24hrs and the cytokine concentrations of the supernatants assayed by Multiplex-ELISA (*n* = 10 (Tc), *n* = 3 (Th)), mean ± SEM.

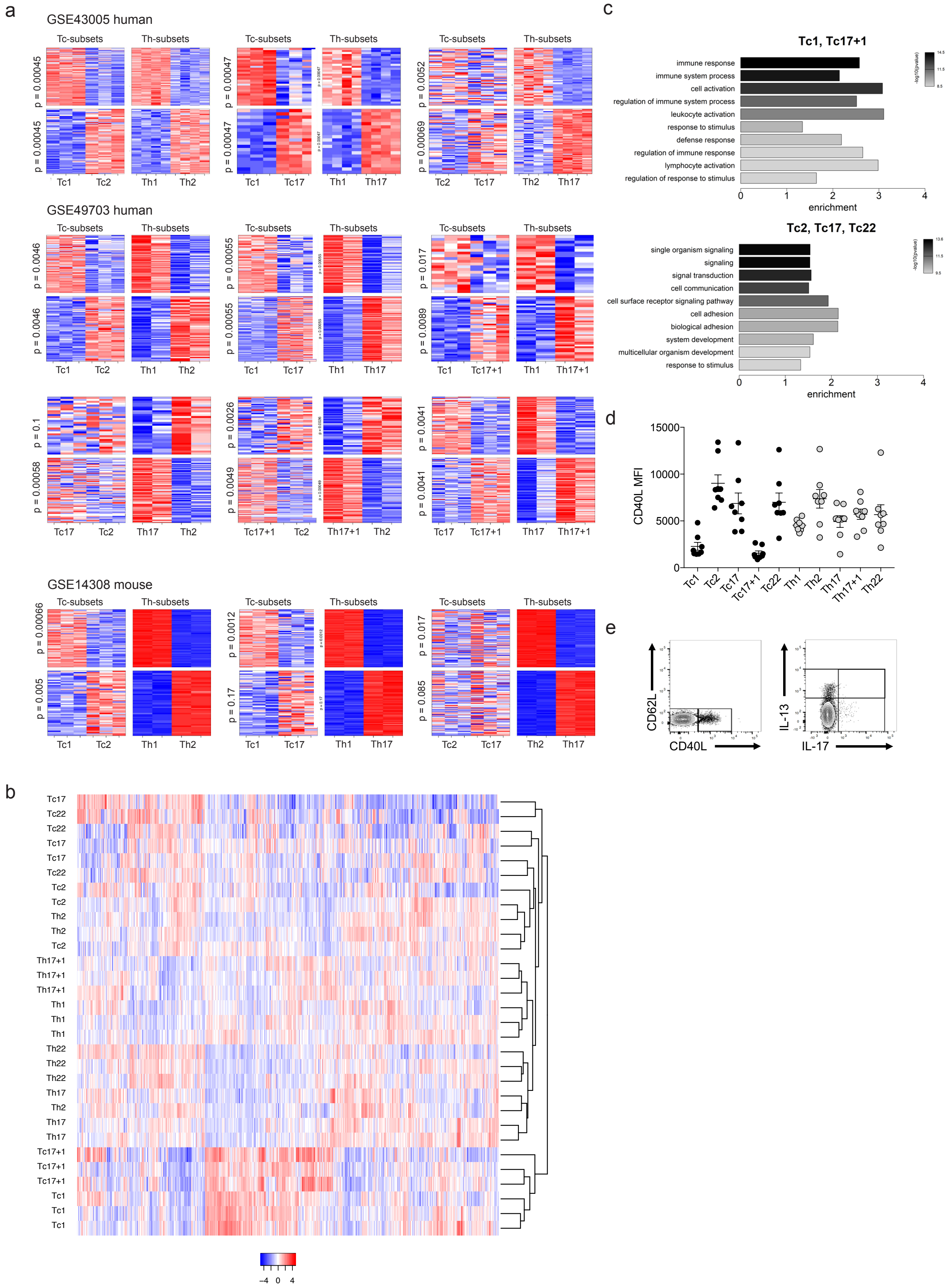
a
GSE43005 human
Tc-subsets
Th-subsets
p = 0.00045
p = 0.00047
p = 0.0052
p = 0.00069
Tc1
Tc2
Th1
Th2
Tc17
Th17
GSE49703 human
p = 0.0046
p = 0.00055
p = 0.017
p = 0.0089
Tc17+1
Th17+1
p = 0.1
p = 0.00058
p = 0.0026
p = 0.0049
p = 0.0041
GSE14308 mouse
p = 0.00066
p = 0.005
p = 0.0012
p = 0.17
p = 0.085
b
Tc22
Th22
−4 0 4
c
Tc1, Tc17+1
immune response
immune system process
cell activation
regulation of immune system process
leukocyte activation
response to stimulus
defense response
regulation of immune response
lymphocyte activation
regulation of response to stimulus
enrichment
Tc2, Tc17, Tc22
single organism signaling
signaling
signal transduction
cell communication
cell surface receptor signaling pathway
cell adhesion
biological adhesion
system development
multicellular organism development
response to stimulus
d
CD40L MFI
15000
10000
5000
0
e
CD62L
CD40L
IL-13
IL-17

Supplementary Figure 2: Gene expression of Tc- and Th-cell subsets.

a, Differentially expressed genes among Th-cell subsets of the GEO datasets GSE43005 (human), GSE49703 (human) and GSE14308 (mouse) (log2 fold change ≥ 1) were calculated and their distribution among differentially expressed genes of Tc-cell subsets tested (Kolmogornov-Smirnov test). The upper boxes of the heatmaps indicate upregulated, the lower boxes downregulated genes respectively. **b,** Heatmap of top 1000 most variably expressed genes after data normalization (number of donors = 3). **c,** Top 10 gene ontology terms from overrepresentation analysis (ORA) of genes upregulated in Tc1/Tc17+1 or Tc2/Tc17/Tc22 cells respectively. **d,** Mean fluorescent intensity (MFI) of CD40L expressing cells among sorted and polyclonally activated Tc- (black circles) and Th- (grey circles) cell subsets (*n* = 8), mean ± SEM. **e,** Representative gating for CD40L, IL-17 and IL-13 in murine spleen derived dump$^-$ CD3$^+$CD44$^+$CD8$^+$ memory T cells upon polyclonal activation and intracellular staining.

a

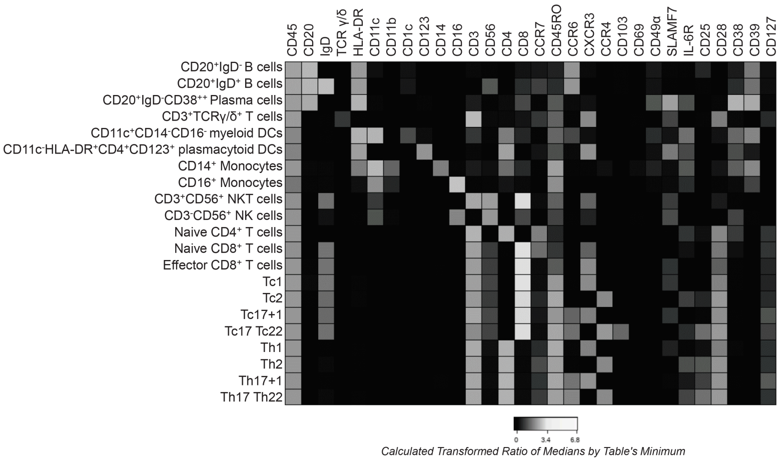

b

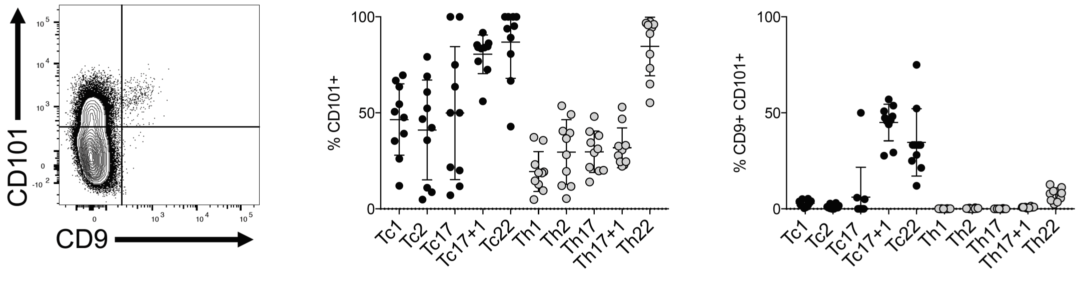

Supplementary Figure 3: CyTOF based screening for T$_{RM}$ markers

a, Heatmap of median marker intensities of indicated markers in the different PBMC derived subsets measured by Mass Cytometry. **b,** Coexpression of CD101 and CD9 among PBMC derived lymphocytes (left). Frequencies of CD101$^+$ and CD9$^+$CD101$^+$ cells among pregated Tc- (black circles)/Th- (grey circles) cell subsets respectively (right). Mean ± SEM.

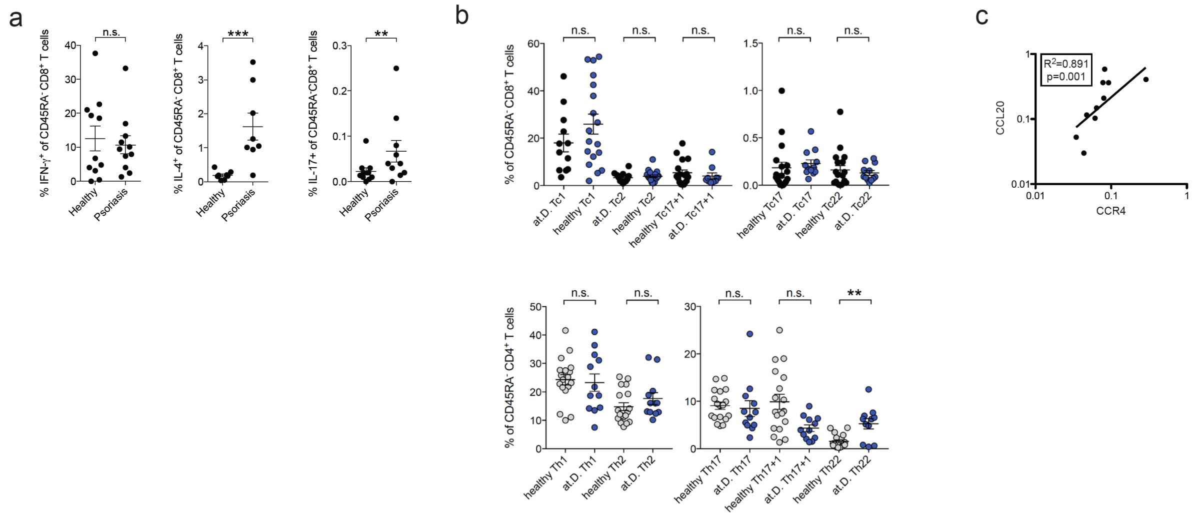

Supplementary Figure 4: CD8$^+$ memory T cells in skin diseases.

a, Cytokine expression of polyclonally activated CD45RA$^-$CD8$^+$ T cells isolated from peripheral blood of healthy donors or patients with psoriasis (n = 11). Mean ± SEM. Students t -test. * $P < 0.05$, ** $P < 0.01$, *** $P < 0.001$. **b,** Frequencies of Tc- (black circles) and Th- (grey circles) cell subsets of healthy donors or patients with atopic dermatitis (at.D.) (blue circles) (n = 13). Mean ± SEM. Students t -test. * $P < 0.05$, ** $P < 0.01$, *** $P < 0.001$. **c,** Correlation of mRNA levels of CCR4 and CCL20 in psoriatic lesions (n = 10).

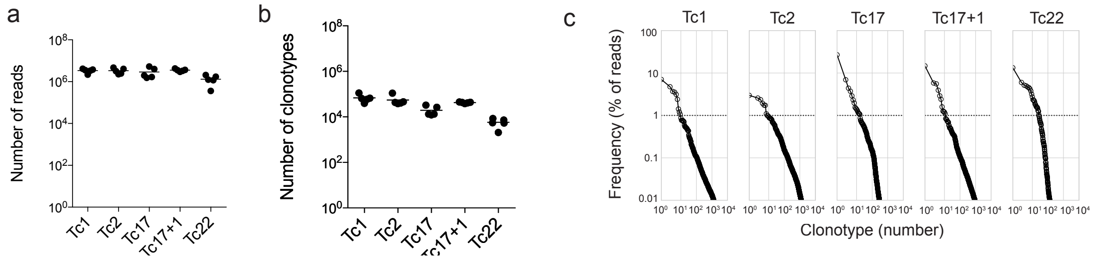

Supplementary Figure 5: Number of reads /clonotypes and their representative distribution.

a, Number of reads in TCR-Sequencing of sorted CD8^{+} T-cell subsets ($n = 5$). Mean. **b,** Number of clonotypes in indicated T-cell subsets ($n = 5$). Mean. **c,** Representative frequency of reads distribution of clonotypes in the different Tc-cell subsets of one donor.

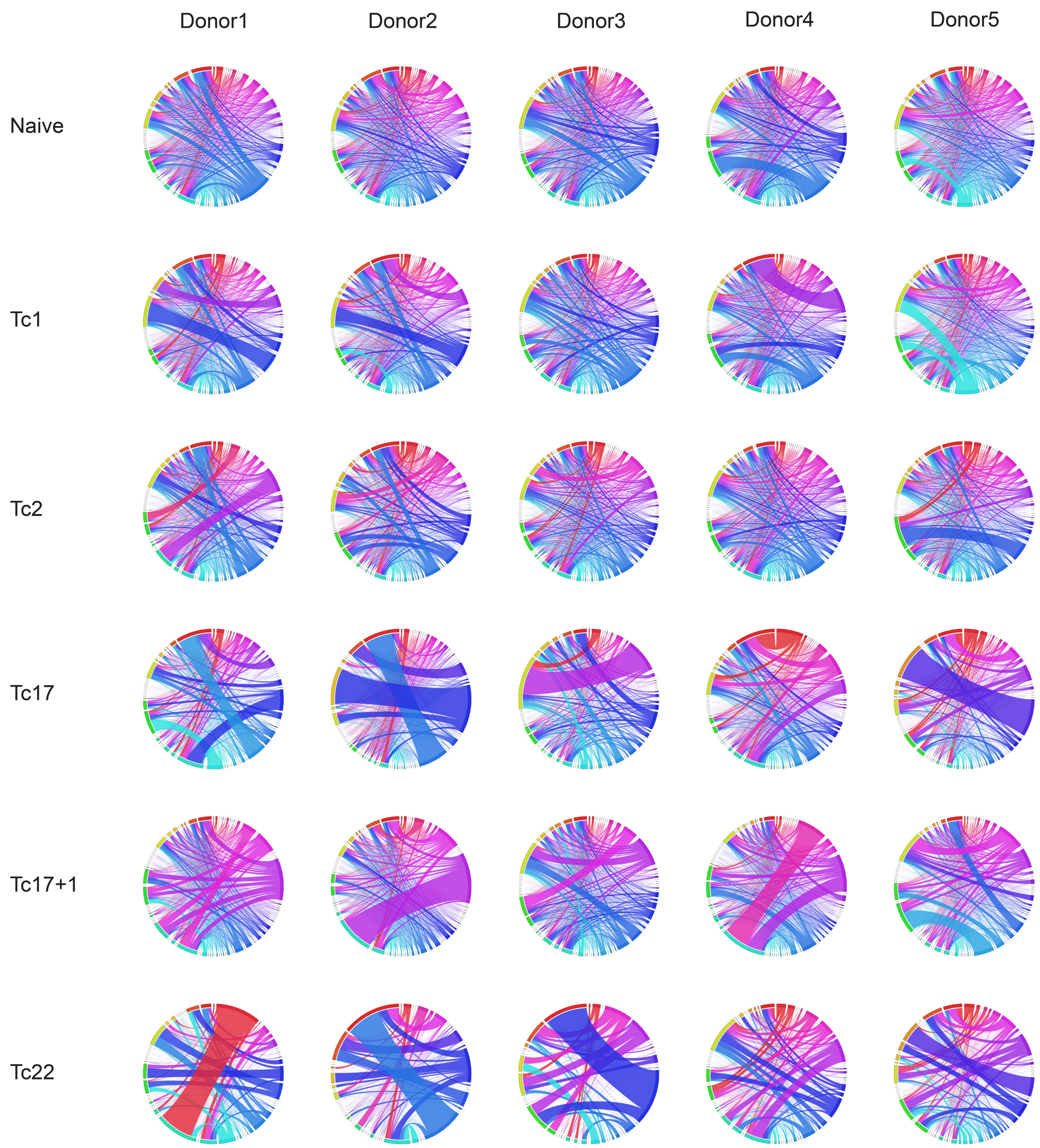
Donor1
Donor2
Donor3
Donor4
Donor5
Naive
Tc1
Tc2
Tc17
Tc17+1
Tc22

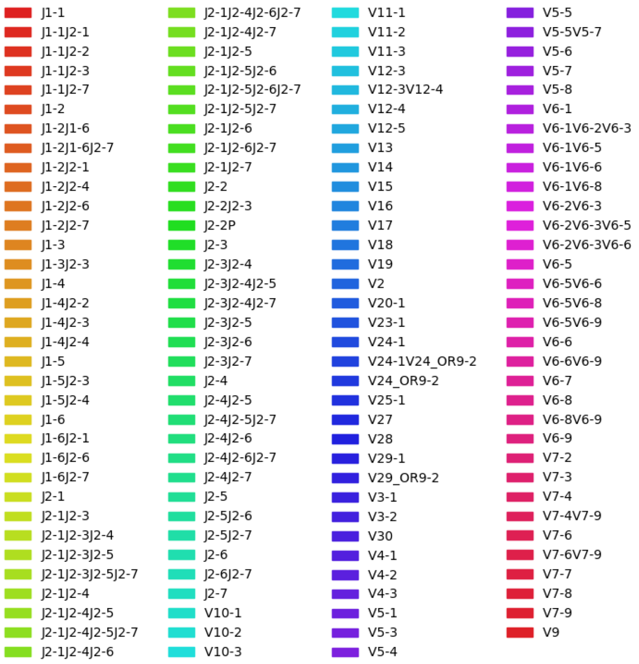

Supplementary Figure 6: TCR-Vβ and -Jβ usage of the Tc-cell subsets.

Circos plots display the combinations and the frequency of Vβ- and Jβ-usage contributing to the clonal repertoire of sorted naïve CD45RA^{+}CCR7^{+} CD8^{+} T cells and memory CD8^{+} T-cell subsets.

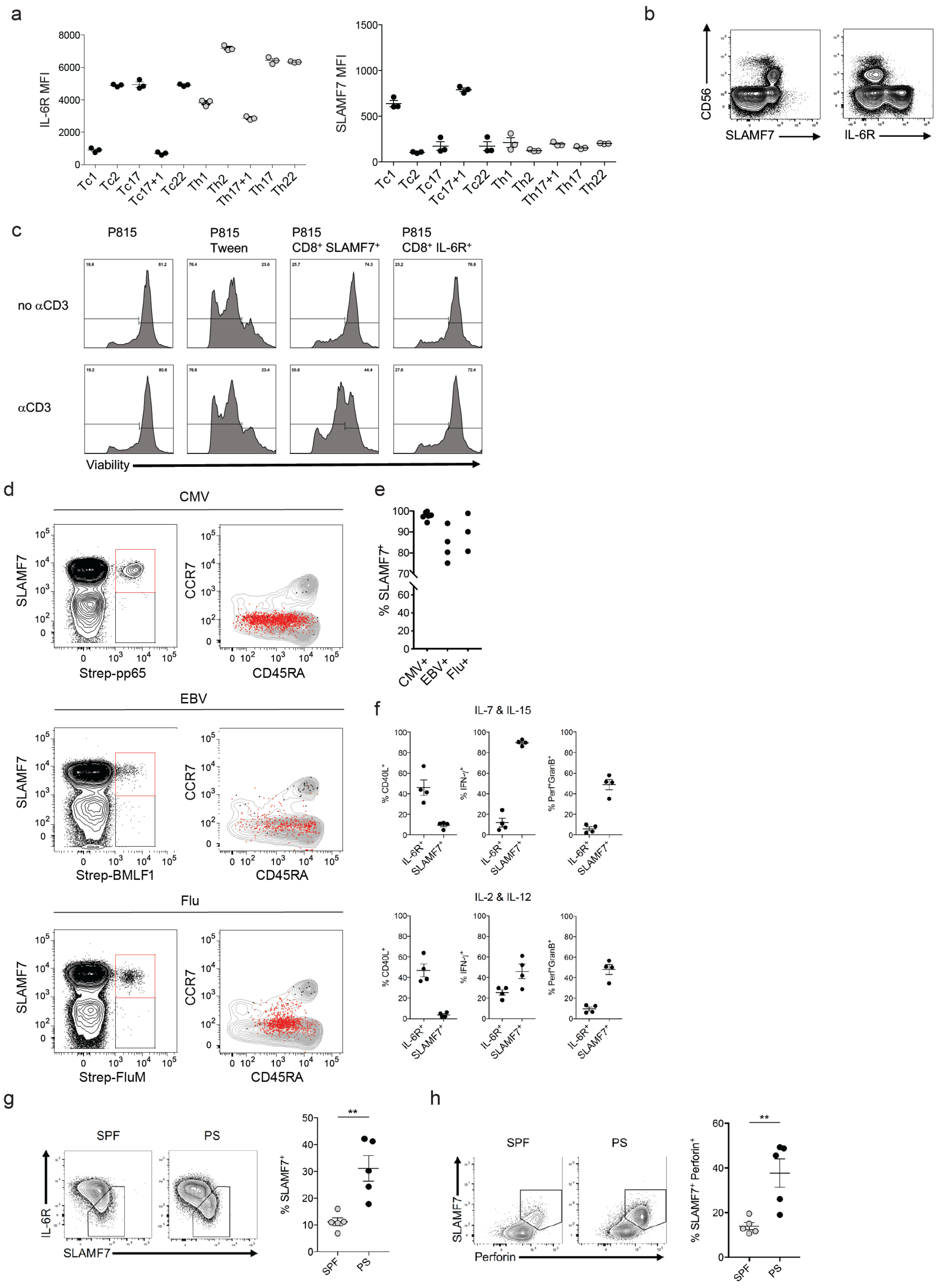

Supplementary Figure 7: Features of SLAMF7 versus IL-6R expressing cells.

a, MFI of IL-6R and SLAMF7 among human naïve CD45RA^{+}CCR7^{+}, EMRA CD45RA^{+}CCR7^{-} and memory CD45RA^{-}CD8^{+} (black circles) and CD4^{+} (grey circles) T-cell subsets. **b,** Representative dot plot of CD56 co-expression with SLAMF7 and IL-6R among

lymphocytes. **c,** Representative histogram of T cell mediated redirected lysis of uncoated or α-CD3 coated p815 cell line at an E:T ratio of 10:1. **d,** Left dot plots: Streptamer and SLAMF7 co-expression of indicated specificity (CMV pp65, EBV BMLF1, Flu M) among $CD8^+$ T cells. Right dot plots: Overlay of CCR7 and CD45RA expression of $Streptamer^+SLAMF7^+$ (red dots) or $Streptamer^+SLAMF7^-$ (black dots) cells with $Streptamer^-$ cells indicates naïve, CM, EM or EMRA phenotype. **e,** Summary of frequencies of $SLAMF7^+$ cells among $Streptamer^+$ $CD8^+$ T cells of n =6 (CMV), n = 4 (EBV) and n = 3 (Flu) different donors. **f,** Sorted $SLAMF7^+$ or $IL\text{-}6R^+$ memory $CD8^+$ T cells were stimulated with plate bound α-CD3/α-CD28 for 2d and cultivated in the presence of either IL-7/IL-15 or IL-2/IL-12 for 7d in total. Upon restimulation with PMA/Iono in the presence of BrefA (6hrs), the expression of CD40L, IFN-γ and GranzymeB/Perforin was assessed by intracellular staining. **g, h,** Representative dot plot and summarized co-expression of SLAMF7 with IL-6R among spleen derived $dump^-CD3^+CD44^+$ $CD8^+$ memory T cells (**g**) and SLAMF7 with Perforin among spleen derived $dump^-CD3^+$ total $CD8^+$ T cells in specific pathogen free (SPF, grey circles) and pet shop (PS, black circles) mice (**h**). Mean ± SEM. Students t -test. * $P < 0.05$, ** $P < 0.01$, *** $P < 0.001$.

a

Patient Data	Number of donors	Gender (m/f)	Age	Disease severity
**Psoriasis**	12	(11/1)	mean 51.1 (S.D.10.3)	PASI SCORE mean 6.4 (S.D. 8.5)
**Atopic Dermatitis**	8	(6/2)	mean 36.7 (S.D.15.5)	SCORAD mean 46.2 (S.D. 20.5)
**Healthy**	18	(14/4)	mean 38.3 (S.D. 6.2)	

b

Patient Data	Number of donors	Gender (m/f)	Age	Disease severity
**Psoriasis**	9	(6/3)	mean 51.19 (S.D. 13.33)	9 severe
**Atopic Dermatitis**	7	(2/5)	mean 26.91 (S.D. 11.92)	6 severe, 1 medium
**Healthy**	10	(9/1)	mean 26.4 (S.D. 12.85)	

Supplementary Table 1: Patient information

a, b, Numbers, gender, age and disease severity of PBMC (**a**) and skin biopsy (**b**) donors
