# Supplementary material for: SLAMF7 and IL-6R define distinct cytotoxic versus helper memory CD8+ T cells
Source: Nat Commun. 2020 Dec 11;11:6357. doi: 10.1038/s41467-020-19002-6 (PMC7733515; doi:10.1038/s41467-020-19002-6)
Supplement: Supplementary file 3 — Reporting Summary [file 41467_2020_19002_MOESM3_ESM.pdf]

natureresearch

Corresponding author(s): Lucie Loyal, Andreas Thiel

Reporting Summary

Nature Research wishes to improve the reproducibility of the work that we publish. This form provides structure for consistency and transparency in reporting. For further information on Nature Research policies, see Authors & Referees and the Editorial Policy Checklist.

Statistical parameters

When statistical analyses are reported, confirm that the following items are present in the relevant location (e.g. figure legend, table legend, main text, or Methods section).

n/a	Confirmed	
☐	☒	The exact sample size (*n*) for each experimental group/condition, given as a discrete number and unit of measurement
☐	☒	An indication of whether measurements were taken from distinct samples or whether the same sample was measured repeatedly
☐	☒	The statistical test(s) used AND whether they are one- or two-sided<br>*Only common tests should be described solely by name; describe more complex techniques in the Methods section.*
☒	☐	A description of all covariates tested
☐	☒	A description of any assumptions or corrections, such as tests of normality and adjustment for multiple comparisons
☐	☒	A full description of the statistics including central tendency (e.g. means) or other basic estimates (e.g. regression coefficient) AND variation (e.g. standard deviation) or associated estimates of uncertainty (e.g. confidence intervals)
☒	☐	For null hypothesis testing, the test statistic (e.g. *F*, *t*, *r*) with confidence intervals, effect sizes, degrees of freedom and *P* value noted<br>*Give P values as exact values whenever suitable.*
☒	☐	For Bayesian analysis, information on the choice of priors and Markov chain Monte Carlo settings
☒	☐	For hierarchical and complex designs, identification of the appropriate level for tests and full reporting of outcomes
☒	☐	Estimates of effect sizes (e.g. Cohen's *d*, Pearson's *r*), indicating how they were calculated
☐	☒	Clearly defined error bars<br>*State explicitly what error bars represent (e.g. SD, SE, CI)*

Our web collection on statistics for biologists may be useful.

Software and code

Policy information about availability of computer code

Data collection	Human reference genome (GRCh37), Ensembl, http://feb2014.archive.ensembl.org/index.html
Data analysis	FlowJo Version 9.9.4 Tree Star<br>GraphPad Prism Version 7.0 GraphPad<br>Bowtie2 Langmead and Salzberg, 2012 http://bowtie-bio.sourceforge.net/bowtie2/index.shtml<br>fastQC Babraham Bioinformatics https://www.bioinformatics.babraham.ac.uk/projects/fastqc/<br>R version 3.3.3 The R Foundation https://cran.r-project.org/<br>R Core Team (2017). R: A language and environment for statistical computing. R Foundation for Statistical Computing, Vienna, Austria.<br>R package "sva" version 3.22.0 (Leek et al., 2012) https://cran.r-project.org/<br>topGO version 2.26.0 (Carlson et al., 2016). org.Hs.eg.db: Genome wide annotation for Human)<br>Imseq v1.1.0 Charité Universitätsmedizin Berlin http://www.imtools.org (Kuchenbecker et al., 2015)<br>Python 3.6 Python Software Foundation https://www.python.org<br>Matplotlib 2.0.2 The MathWorks, Inc. https://matplotlib.org (Hunter, 2007)<br>Pandas 0.20.3 Pandas Developers http://pandas.pydata.org<br>McKinney, W. Data Structures for Statistical Computing in Python, Proceedings of the 9th Python in Science Conference, 51-56 (2010).<br>NumPy 1.13.1 NumPy Developers http://www.numpy.org (Van Der Walt et al., 2011)<br>SciPy 0.19.1 SciPy Developers http://www.scipy.org/ Jones E., Oliphant E., Peterson P., et al. SciPy: Open Source Scientific Tools for

Python, 2001.
Circos Genome Data Visualization http://circos.ca/intro/genomic_data/

For manuscripts utilizing custom algorithms or software that are central to the research but not yet described in published literature, software must be made available to editors/reviewers upon request. We strongly encourage code deposition in a community repository (e.g. GitHub). See the Nature Research guidelines for submitting code & software for further information.

Data

Policy information about availability of data

All manuscripts must include a data availability statement. This statement should provide the following information, where applicable:
- Accession codes, unique identifiers, or web links for publicly available datasets
- A list of figures that have associated raw data
- A description of any restrictions on data availability

RNA-Sequencing data is deposited and accessible at NCBI GEO Expression Omnibus under GSE115103

Field-specific reporting

Please select the best fit for your research. If you are not sure, read the appropriate sections before making your selection.

[x] Life sciences [ ] Behavioural & social sciences [ ] Ecological, evolutionary & environmental sciences

For a reference copy of the document with all sections, see nature.com/authors/policies/ReportingSummary-flat.pdf

Life sciences study design

All studies must disclose on these points even when the disclosure is negative.

Sample size	Majority of data is descriptive with clear differences between the groups. For conclusions from patient samples, sample numbers were fit to data reproducibility of healthy control group in order to exclude unspecific effects by inter-human variances.
Data exclusions	No data was excluded
Replication	Flow stainings were repeated in independent experiments, partially not shown here
Randomization	All experiments were conducted from blood samples delivered by a blood bank without further information. Patients were selected according to medical recordings by clinicans and controlled by age and gender matching healthy.
Blinding	Patient samples were collected at different days from clinics and measured alongside healthy controls using half-blind approach by using acronyms which where associated to patient/control after the measurement. Severity scores were collected and aligned after analysis was conducted.

Reporting for specific materials, systems and methods

Materials & experimental systems

n/a	Involved in the study	
x		Unique biological materials
	x	Antibodies
x		Eukaryotic cell lines
x		Palaeontology
	x	Animals and other organisms
	x	Human research participants

Methods

n/a	Involved in the study	
x		ChIP-seq
	x	Flow cytometry
x		MRI-based neuroimaging

Antibodies

Antibodies used	CD4 (Clone RPA-T4) Biolegend Cat# 300526, RRID:AB_493743<br>CD8 (Clone RPA-T8) BD Biosciences Cat# 560775, RRID:AB_1937333<br>CD45RA (Clone HI100) Biolegend Cat# 304126, RRID:AB_10708879<br>CCR7 (Clone G043H7) Biolegend Cat# 353206, RRID:AB_10916389<br>CCR6 (Clone G034E3) Biolegend Cat# 353420, RRID:AB_2561449<br>CCR4 (Clone L291H4) Biolegend Cat# 359406, RRID:AB_2562391<br>CCR10 (Clone 6588-5) Biolegend Cat# 341504, RRID:AB_1595446<br>CXCR3 (Clone G025H7) Biolegend Cat# 353712, RRID:AB_10962948<br>SLAMF7 (CD319) (Clone 162.1) Biolegend Cat# 331816, RRID:AB_2565237<br>IL-6R (Clone UV4) Biolegend Cat#352804, RRID: AB_10900066<br>GranzymeB (Clone GB11) BD Biosciences Cat# 561999, RRID:AB_10897997<br>Perforin (Clone B-D48) Abcam Cat# ab47226<br>IFNγ (Clone B27) Biolegend Cat# 506516, RRID:AB_961351<br>CD40L (Clone 5C8) Miltenyi Biotec Cat# 130-092-290<br>IL-4 (Clone MP4-25D2) Biolegend Cat# 500810, RRID:AB_315129<br>IL-17A (Clone BL168) Biolegend Cat# 512320, RRID:AB_10613103<br>IL-22 (Clone 22URTI) Thermo Fisher Scientific Cat# 48-7229-42, RRID:AB_11150956<br>GATA3 (Clone REA174) Miltenyi Biotec Cat# 130-100-651<br>AHR (Clone FF3399) Thermo Fisher Scientific Cat# 46-9854-41, RRID:AB_2573905<br>CD57 (Clone HCD57) Biolegend Cat# 322305<br>CD28 (Clone CD28.2) Biolegend Cat# 302921<br>CD103 (Clone Ber-ACT8) Biolegend Cat#350228, RRID:AB_2734362<br>CD101 (Clone BB27) Biolegend Cat# 331014, RRID:AB_2716109<br>CD9 (Clone HI9a) Biolegend Cat#312114, RRID:AB_2728254<br>CD69 (FN50) Biolegend Cat#310920, RRID: AB_493667<br>CD3 (17A2) Biolegend Cat# 100240, RRID:AB_256342<br>CD4 (RM4-5) Biolegend Cat#100528, RRID:AB_312729<br>CD8 (53-6.7) Miltenyi Biotec Cat#130-102-805, RRID:AB_2659890<br>CD62L (MEL-14) Thermo Fisher Scientific Cat# 11-0621-81, RRID:AB_465108<br>CD44 (IM7) Biolegend Cat# 103026, RRID:AB_493713<br>CD19 (6D5) Biolegend Cat# 115523, RRID:AB_439718<br>NK1.1 (PK136) Biolegend Cat# 108732, RRID:AB_2562218<br>IL-6R (D771517) Biolegend Cat# 115806, RRID:AB_313677<br>SLAMF7 (4G2) Biolegend Cat# 152004, RRID:AB_2632675<br>IL-17 (TC11-18H10.1) Biolegend Cat# 506928, RRID:AB_2629787<br>IL-13 (eBio13A) Thermo Fisher Scientific Cat# 47-7133-82, RRID:AB_2716964<br>CD40L (MR1) Miltenyi Biotec Cat# 130-102-454, RRID:AB_266112<br>Perforin (S16009A) Thermo Fisher Scientific Cat# 12-9392-80, RRID:AB_46624<br>CD45 (HI30) Fluidigm Cat# 3089003, RRID:AB_ 2661851<br>CD1c (L161) BioLegend Cat# 331502, RRID:AB_1088995<br>TCRγ/δ (B1) BioLegend Cat# 331202, RRID:AB_1089222<br>CD69 (FN50) BioLegend Cat# 310902, RRID:AB_314837<br>CD11b (ICRF44) BioLegend Cat# 301302, RRID:AB_314154<br>CCR4 (L291H4) BioLegend Cat# 359402, RRID:AB_2562364<br>CD20 (2H7) Fluidigm Cat# 3147001, RRID: n/a<br>CD127 (A019D5) BioLegend Cat# 351302, RRID:AB_10718513<br>CD123 (6H6) Fluidigm Cat# 3151001, RRID:AB_2661794<br>CD103 (Ber-ACT8) BioLegend Cat# 350202, RRID:AB_10639864<br>CD14 (M5E2) BioLegend Cat# 301802, RRID:AB_314184<br>CXCR3 (G025H7) Fluidigm Cat# 3156004B, RRID:AB_2687646<br>CD11c (Bu15) Fluidigm Cat# 3159001, RRID:AB_2661800<br>CD28 (CD28.2) Fluidigm Cat# 3160003, RRID: n/a<br>CD8 (GNM/134D7) purchased from DRFZ<br>IgD (IA6-2) BioLegend Cat# 348202, RRID:AB_10550095<br>CD56 (NCAM16.2) Fluidigm Cat# 3149021, RRID: n/a<br>CD45RO (UCHL1) Fluidigm Cat# 3164007, RRID: n/a<br>CCR6 (G034E3) BioLegend Cat# 353402, RRID:AB_10918625<br>CD3 (UCHT1) BioLegend Cat# 300402, RRID:AB_314056<br>CCR7 (G043H7) BioLegend Cat# 353202, RRID:AB_10945157<br>CD4 (TT1) purchased from DRFZ<br>CD25 (2A3) Fluidigm Cat# 3169003, RRID:AB_2661806<br>SLAMF7 (162.1) BioLegend Cat# 331802, RRID:AB_961330

CD38 (HIT2) BioLegend Cat# 303502, RRID:AB_314354
CD39 (A1) BioLegend Cat# 328202, RRID:AB_940438
CD49a (TS2/7) (BioLegend Cat# 328302, RRID:AB_1236385
IL-6R (UV4) BioLegend Cat# 352802, RRID:AB_11204434
HLA-DR (L243) BioLegend Cat# 307602, RRID:AB_314680
CD16 (3G8) Fluidigm Cat# 3209002, RRID: n/a

Validation

All antibodies are established, well described and published elsewhere. Informations are accessible on the manufacturers websites under Cat# or RRID numbers.

Animals and other organisms

Policy information about studies involving animals; ARRIVE guidelines recommended for reporting animal research

Laboratory animals	Female C57BL/6J mice purchased from Charles River Laboratories, held under SPF conditions. Mice were anesthetized with Isoflurane (AbbVie) prior to sacrificing by cervical dislocation at the age of 12-18 weeks.
Wild animals	Female wild mice were purchased from PetShops in Berlin, Germany. Mice were anesthetized with Isoflurane (AbbVie) prior to sacrificing by cervical dislocation at the age of 12-18 weeks.
Field-collected samples	-

Human research participants

Policy information about studies involving human research participants

Population characteristics	Psoriasis patients were diagnosed psoriasis >10y and treated with anti-TNF, Fumarates, MTX, anti-p40 or combinations. Atopic dermatitis patients were diagnosed according to their serum IgE levels (median 756 kU/L,(IQR 3987;194) and sensitized against > common aeroallergens. Age and gender matches without reports of skin irritations were selected as healthy controls.
Recruitment	Patients were recruited by clinicians according to their appearance in official consulting hours accompanied by active disease status/matching medical recordings. Healthy controls were selected random by picking people with matching age and gender.

Flow Cytometry

Plots

Confirm that:

- [x] The axis labels state the marker and fluorochrome used (e.g. CD4-FITC).
- [x] The axis scales are clearly visible. Include numbers along axes only for bottom left plot of group (a 'group' is an analysis of identical markers).
- [x] All plots are contour plots with outliers or pseudocolor plots.
- [x] A numerical value for number of cells or percentage (with statistics) is provided.

Methodology

Sample preparation	Human blood was obtained from buffy coats (DRK Blutspendedienst Ost) or from healthy volunteers. Blood from psoriasis and atopic dermatitis patients was obtained by K. Wolk, R. Sabat and G. Heine, Charité, after informed consent.<br>Peripheral blood mononuclear cells (PBMCs) were separated from heparinized whole blood by density centrifugation (Biocoll, Biochrom).<br>Skin biopsies were obtained obtained by K. Wolk, R. Sabat from healthy human skin and lesions of psoriasis and atopic dermatitis, after informed consent and disrupted mechanically and passed through a 70µm cell strainer to obtain single-cell suspension.
Instrument	Samples were measured at LSRII (BD) or sorted at FACS Aria (BD).
Software	Data was collected using FACSDiva software (BD) and analyzed with FlowJo (TreeStar). Statistics were conducted using GraphPad Prism.
Cell population abundance	Cell subsets were sorted with >99% purity as controlled by remeasurement of sorted populations.
Gating strategy	Populations as indicated in figure legends were identified by lymphocyte gate in FSC-A/SSC-A, doublet exclusion by FSC-W/FSC-H and SSC-W/SSC-H. Dead cells were excluded by co-staining with live/dead markers (see Supplementary Figure 8) . Following gatings were conducted using markers as described in figure legends.

- [x] Tick this box to confirm that a figure exemplifying the gating strategy is provided in the Supplementary Information.
